# Supplementary material for: Multiplexed detection of SARS-CoV-2 and other respiratory infections in high throughput by SARSeq
Source: Nat Commun. 2021 May 25;12:3132. doi: 10.1038/s41467-021-22664-5 (PMC8149640; doi:10.1038/s41467-021-22664-5)
Supplement: Supplementary file 1 — Supplementary Information [file 41467_2021_22664_MOESM1_ESM.pdf]

Supplemental Figures and Table

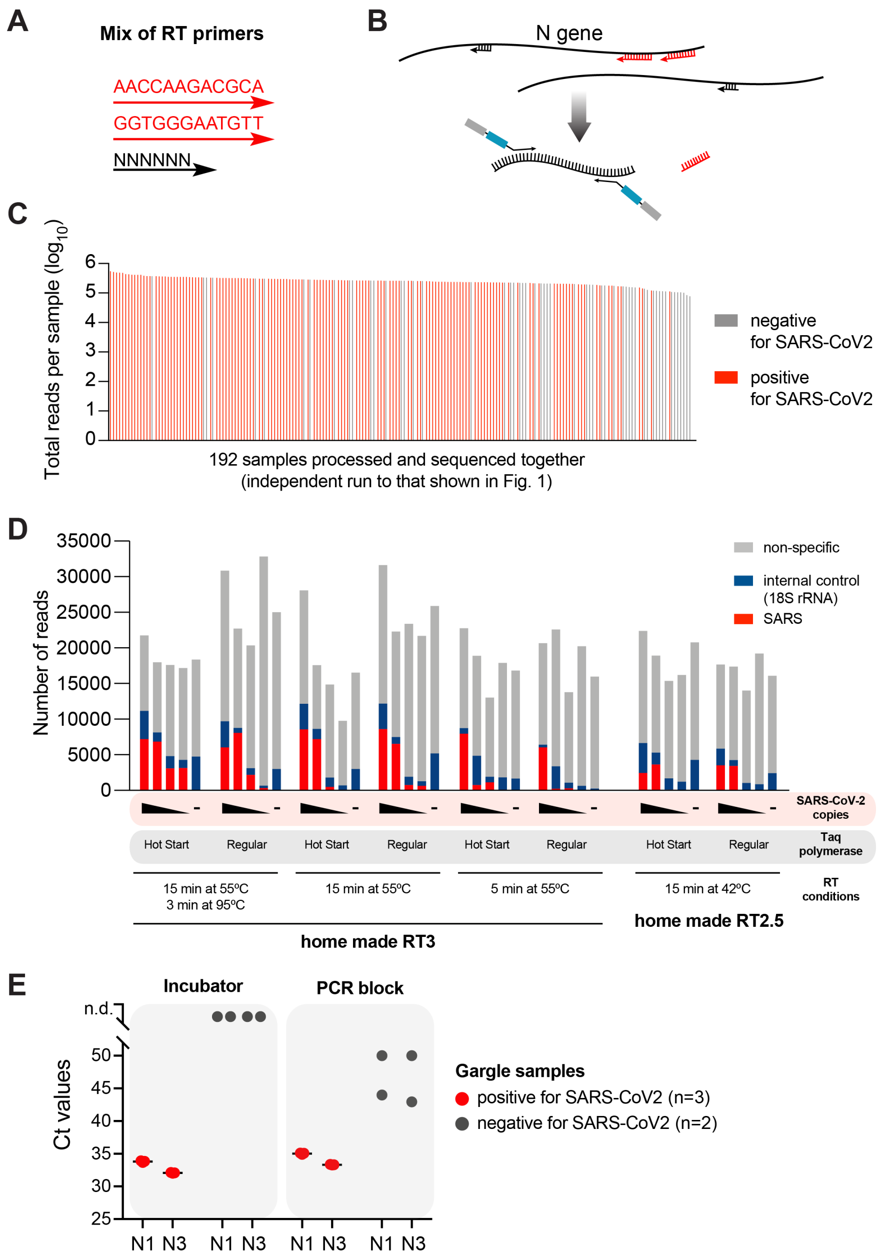

Suppl. Fig. 1. Two-step RT-PCR strategy enables specificity and even read distribution. A. The RT primer mix contains two N-gene specific 12-mers (actual sequences shown) in addition to random hexamers. **B.** Scheme of the RT priming sites relative to the specific PCR primers. For SARS-CoV-2 the 12-mers generate a cDNA into which the PCR primers are nested. **C.** Total reads per sample across a set of 192 samples that contain several negative samples as well as positive samples with titers spanning 4 orders of magnitude. Our 2-step RT-PCR strategy followed by end-point PCR lead to a very even distribution of NGS reads per sample independent of viral titer, ensuring equal representation on the sequencing flow cell. **D.** Amplicon counts measured by NGS after RT and PCR using different conditions, as indicated, on gargle samples from a healthy individual mixed 1:1 with QuickExtract and spiked with a serial dilution of synthetic SARS-CoV-2 RNA (5000, 500, 50, 5, 0 copies). **E.** Quantitative RT-PCR analysis (based on fluorescent DNA dye, Promega) on positive and negative gargle samples, for which the RT reaction was conducted for 15 min at 55ºC either in a PCR block or a humid incubator. All data points are shown, n=3 for positive samples, n=2 for negative samples.

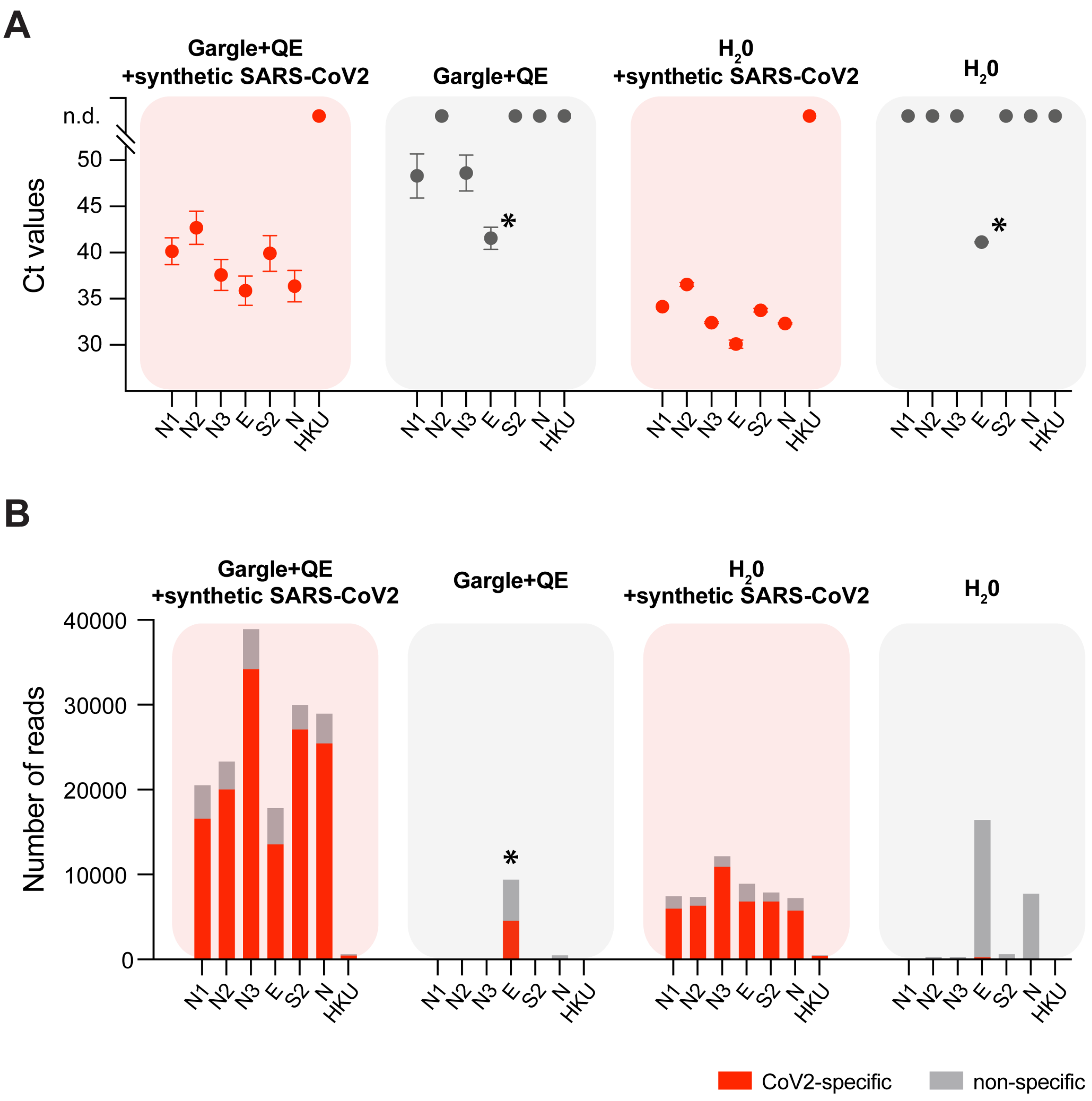

Suppl. Fig. 2. Choice of SARS-CoV-2 specific primers. A. qPCR using a fluorescent DNA dye (Promega) on samples generated by mixing gargle from a healthy individual or water, with 1000 copies of synthetic SARS-CoV-2 RNA. Primers used were based on previous publications[33,35], but were extended at the 5' by the sequences required for NGS, namely i5/i7, a random stagger, and a barcode. Several primer pairs showed a good template dependent generation of double stranded DNA. Mean and standard deviation are shown, n=3. **B.** Number of specific and non-specific reads obtained by SARSeq using seven different primer pairs directed at distinct regions of the SARS-CoV-2 genome (N1, N2, N3 and S2 from the CDC; E from Sarbeco; N from China and HKU). Primers were tested on synthetic SARS-CoV-2 RNA diluted in gargle from a healthy individual or in water. All primer pairs, except for HKU efficiently amplified viral products. Asterisk denotes evident contamination with an E gene amplicon used in other testing efforts.

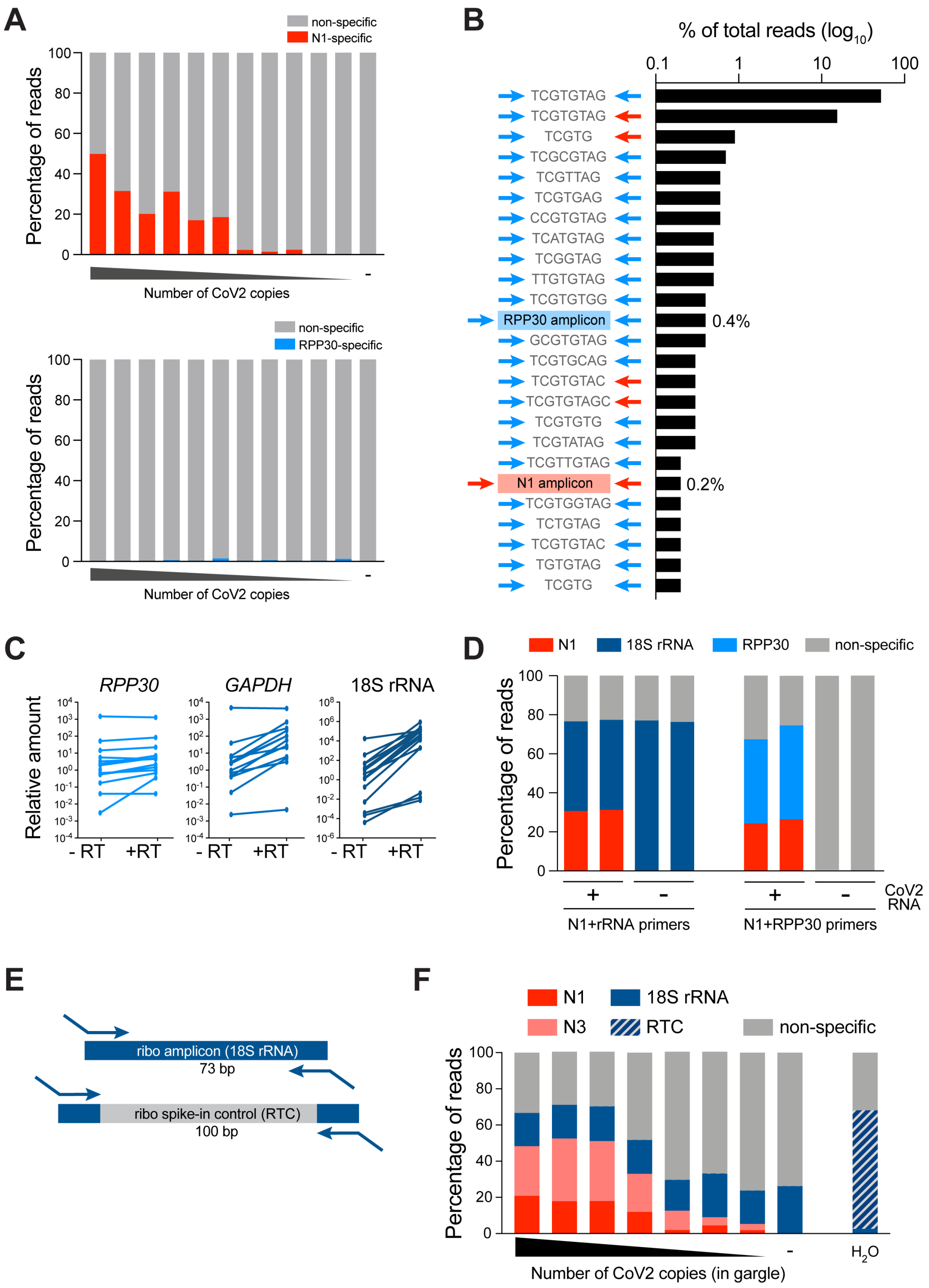

Suppl. Fig. 3. A control primer pair targeting 18S rRNA improves assay performance compared to the widely used *RPP30* primers. A. Percentage of specific and non-specific NGS reads obtained with the SARS-CoV-2 N gene-specific primer pair N1 or the human RNAse P (*RPP30*)-specific primer pair designed by the CDC[35]. RT-PCR was performed on inactivated gargle-QE samples spiked with a range of 5120 to 5 molecules of synthetic template in a two-fold dilution series, as well as 0 molecules. Non-specific amplicons are defined as

amplicons generated by the respective matched primer pairs but with incorrect sequence in between. This experiment was performed in a first-generation buffer system that was further modified in subsequent experiments. **B.** Analysis of all amplicons generated in the pool of conditions shown in A. Non-specific amplicons typically incorporated a short stretch of sequence complementary to some primer sequences and were almost exclusively generated by at least one *RPP30*-specific primer. Specific amplicons add up to <1% of reads in this condition. **C.** qPCR analysis (using a fluorescent DNA dye Promega) of *RPP30* primers and two alternative internal control primer pairs in the presence and absence of reverse transcriptase on RNA purified from gargle of 16 individuals. Ct values are transformed to relative differences. **D.** Read distribution from an NGS experiment similar to A, but with the ribosome amplicon as internal control in comparison to RNAseP, and in the final buffer conditions used for the rest of the experiments (and described in the methods). RT-PCR was performed on inactivated gargle-QE samples with or without synthetic SARS-CoV-2 RNA. Although *RPP30* primers performed well in the presence of synthetic viral RNA, they again did not produce specific reads on gargle alone and therefore do not provide a good measure for sample quality under our assay conditions. **E.** Scheme of the RT control (RTC) spike-in; an amplicon with identical primer binding sites to the ribosomal internal control yet different and longer intermediate sequence was synthesized, cloned, and T7 transcribed. The RTC was added during the RT at 1000 molecules/reaction. The ratio of ribosomal to RTC reads serves as a sample quality measurement. Reactions without reads for the ribosome or the RTC amplicons indicate an inhibited/failed RT-PCR reaction. **F.** Read distribution from an NGS experiment containing three primer pairs (N1, N3 and rRNA as internal control) and with addition of the RTC spike-in. The large fraction of specific reads enables high sensitivity and scalability.

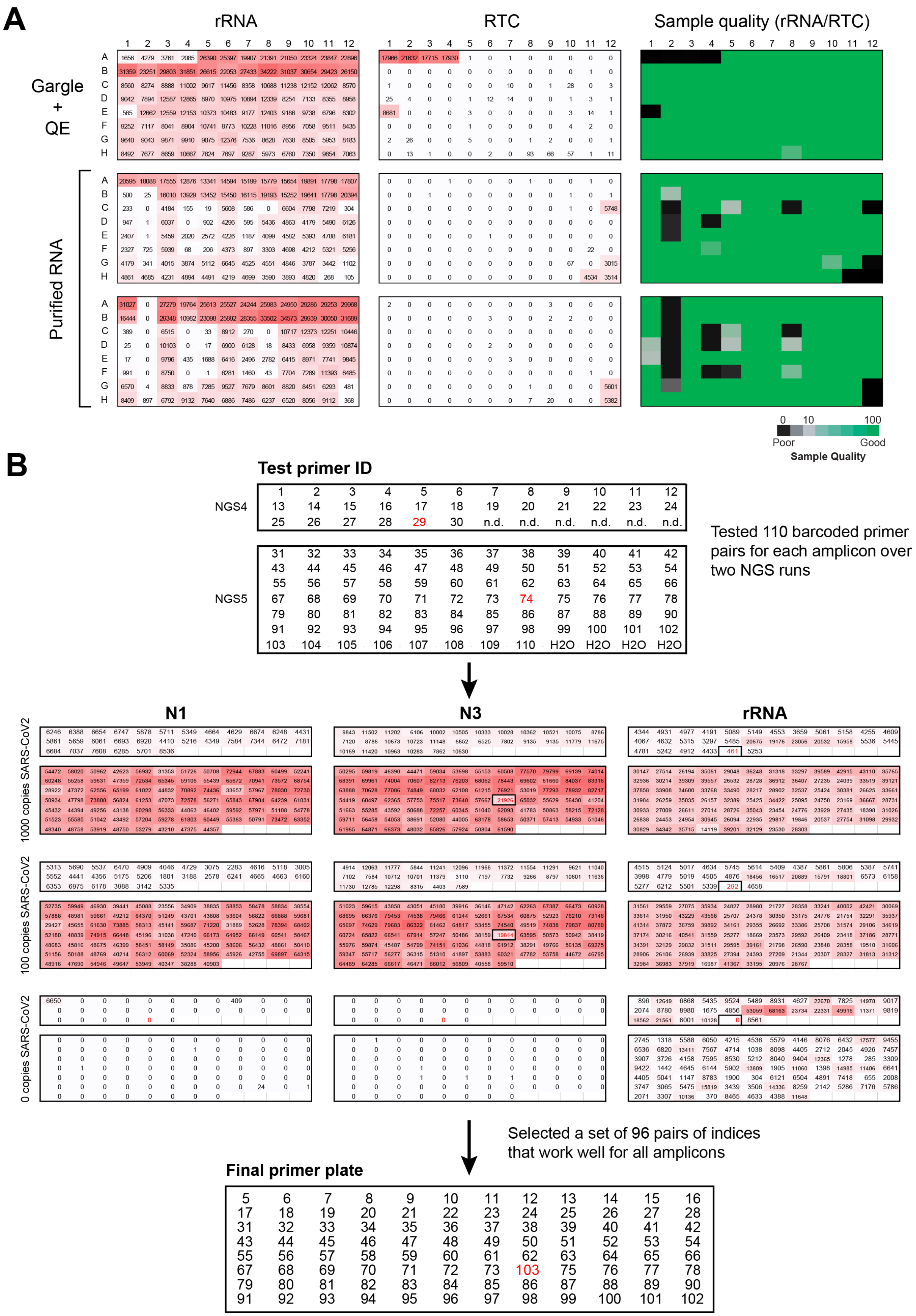
A
rRNA
RTC
Sample quality (rRNA/RTC)
Gargle + QE
Purified RNA
0 10 100
Poor Good
Sample Quality
B
Test primer ID
NGS4
NGS5
Tested 110 barcoded primer pairs for each amplicon over two NGS runs
N1
N3
rRNA
1000 copies SARS-CoV2
100 copies SARS-CoV2
0 copies SARS-CoV2
Selected a set of 96 pairs of indices that work well for all amplicons
Final primer plate

Suppl. Fig. 4. Internal control primers and validation of the amplicon-specific primer pairs containing 96 unique dual indices. A. NGS reads obtained from 2 different sample plates. The plate containing gargle inactivated in QuickExtract generated a good number of reads for almost all samples with few reads in the first four negative control wells (some reads are likely due to rRNA contamination in the enzyme preps), where instead high read counts for the RTC are observed. The other plate contained purified RNA from samples obtained in a clinical setting and was run in duplicates (both are shown). For this plate, frequently both the rRNA and RTC reads failed in certain positions. Given that in absence of RNA the RTC amplicon is expected to amplify very efficiently, we attribute this to the presence of some inhibitor of RT and/or PCR, likely remaining from the RNA purification in these positions. Negative control wells for the latter two plates are positions G12 and H12. **B.** Testing of primer pairs for three amplicons (SARS-CoV-2 N1 and N3, and human ribosome) carrying extensions with 110 unique dual indices. Primer pairs with indices #29 were excluded as they did not efficiently produce the ribosome amplicon; primer pairs with indices #74 were excluded as they did not efficiently produce the N3 amplicon. Primer pairs 1-4 can be used efficiently but were not included in the final set because these indices were frequently used in the lab during initial setup of the method and we wanted to eliminate all possible sources of cross contamination.

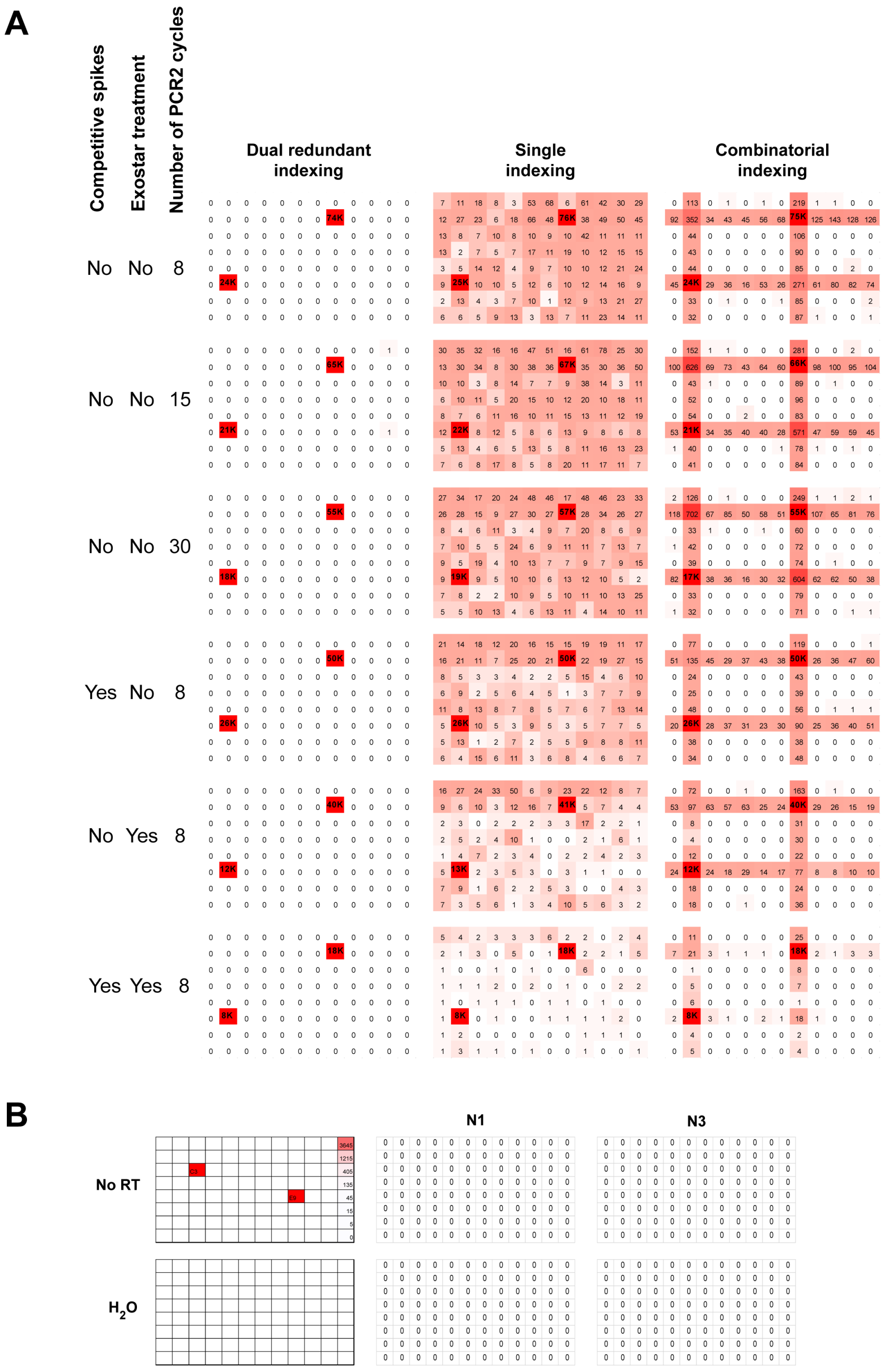

Suppl. Fig. 5. Effect of different measures to suppress sample-index mis-assignments. A. SARS-CoV-2 synthetic RNA was added to positions B8 and F2 only. Assigning sample identities based on single indices or combinatorial indices produces a substantial amount of misassignments. These can be reduced with three independent measures: addition of a competitive spike-in and treatment with Exostar to remove any left-over primers after PCR1, and limiting the number of cycles in PCR2 to prevent recombination between amplicons and their indices. Whereas all these measures help reduce misassignments, adding dual redundant indices (or unique dual indices) completely suppresses this. **B.** Shown are N1 and N3 reads for two negative control plates (-RT and H_2O) that were run together with numerous other positive plates in the same run (shown in **Fig. 3A, C, E**). The fact that we detect 0 reads shows that there is no misassignment of well identities across plates either. This is because each plate is also encoded by redundant/unique dual indices.

A

	N1 amplicon reads									N3 amplicon reads								
	synthetic RNA in H2O			synthetic RNA in QE-HBSS			virions in QE-HBSS			synthetic RNA in H2O			synthetic RNA in QE-HBSS			virions in QE-HBSS		
Ribosomal spike-in (RTC) only – 1 copy/reaction	0	1587	4615	0	7104	8079	18424	4297	0	0	16691	21055	0	40815	20755	44921	3712	0
	0	0	0	0	0	6254	0	22763	22770	0	0	0	0	0	29900	0	46506	53074
	0	0	635	1084	0	0	0	501	3930	0	0	376	1403	0	0	1708	289	0
	0	0	0	0	0	0	0	6349	2001	0	0	0	0	0	0	834	13032	5173
	0	1526	0	0	0	2887	0	3999	0	0	5867	0	7440	0	14679	0	7537	0
	901	0	0	0	1538	1263	7983	1791	3771	4920	0	0	0	6711	10433	11770	3449	6663
	0	0	0	1670	2399	42	0	2526	7116	0	0	0	3133	5973	0	2789	5428	0
	0	0	0	0	0	0	984	0	1580	0	4483	0	0	0	0	0	0	3369
Ribosomal spike-in (RTC) only – 3 copy/reaction	638	1630	4537	3355	2438	0	15315	7913	15461	0	15021	21155	23176	16815	0	48409	39772	25154
	1076	0	835	0	6257	3072	13725	0	3348	9609	0	17414	0	14594	0	36411	38122	18937
	4464	712	112	741	3249	1683	4103	4549	3697	5961	0	177	2043	3512	3062	7219	5732	5900
	240	334	0	1829	0	845	5986	3316	7060	1346	1679	1	4711	2088	759	6446	14598	6102
	3677	2195	0	5646	2557	748	7531	9259	8028	2580	8772	5136	12186	3037	7300	4250	0	11295
	1355	711	0	2902	0	0	4433	7744	10986	4417	6002	0	8282	0	0	889	10241	7573
	2934	2461	424	1032	0	1135	2145	2710	4812	3321	5013	1869	3840	5339	0	6138	9451	3967
	1401	0	2219	1045	2221	5366	13213	9040	11647	5395	0	3621	428	6013	3615	11601	9405	10022
Ribosomal spike-in (RTC) only – 10 copy/reaction	2176	9737	5772	7574	3419	0	9017	7417	6906	6176	23369	22913	28309	23596	9876	22500	31220	24338
	1956	1231	5184	4454	6691	11330	7984	11242	12095	14745	14514	12988	15653	19354	28088	22964	29442	24319
	5110	1849	507	1253	3532	2283	2332	1430	6407	4924	336	673	2279	3880	4232	2381	3679	7801
	2448	3186	1978	2598	3030	1495	3336	3766	4976	4467	6627	5169	5353	5730	2810	3108	10380	4285
	981	1315	2408	5092	3486	1115	7635	4098	5720	1232	2854	8289	7079	8192	5783	3978	6094	7167
	4505	2460	4184	2472	2205	1353	9928	3493	6887	7163	8739	6703	5114	9342	5117	5297	7337	9714
	3055	1578	1466	2910	0	774	2655	2442	6191	4879	2748	1586	4786	0	1341	5337	6899	2716
	1530	5326	969	1917	4342	3446	7355	6923	4933	1735	8381	4407	532	3336	3117	6862	7785	4730

B

	N1 amplicon reads									N3 amplicon reads								
	synthetic RNA in H2O			synthetic RNA in QE-HBSS			virions in QE-HBSS			synthetic RNA in H2O			synthetic RNA in QE-HBSS			virions in QE-HBSS		
N1, N3 and Ribosomal spike-ins – 1 copy/reaction	0	4349	0	950	3138	391	7037	3544	5217	0	12048	15884	16979	20989	0	23169	6577	10455
	0	0	0	0	2941	1757	0	0	2179	0	0	0	5616	19459	0	5246	8132	0
	2759	0	0	509	0	1266	565	676	4768	2084	0	94	1193	0	1442	1328	1620	0
	0	0	0	183	0	0	0	2390	3389	1820	0	0	0	0	0	879	930	2354
	0	1033	0	3249	0	0	655	5826	5624	0	6878	0	9281	0	0	1265	7859	8444
	0	1139	0	0	0	168	5663	915	2020	0	2254	0	0	0	5622	5530	1757	6478
	0	908	634	738	1044	0	2501	561	2044	0	1016	957	1204	1526	0	2843	3206	923
	0	0	0	0	5334	0	4055	7798	0	0	0	625	0	1583	0	3857	6111	0
N1, N3 and Ribosomal spike-ins – 3 copy/reaction	0	0	0	5843	7205	0	11110	7611	2130	0	0	0	23614	25551	0	38179	36931	15644
	0	0	0	1893	2593	0	5775	17769	11247	0	0	0	3431	8359	0	22203	40006	35715
	0	2259	0	0	1803	1023	3034	2425	5185	2432	3244	1048	0	3240	939	5046	3255	6524
	0	1260	864	1127	1905	1027	5791	3628	776	0	2917	2353	3604	3999	1344	5170	10143	5135
	1540	1220	2049	935	0	2927	10326	6431	6112	4772	5261	5285	2862	0	7965	6828	4936	5926
	0	346	0	1966	2908	0	3820	6536	1965	0	5549	4777	5643	7471	1	1630	7812	6723
	1264	983	0	0	1603	816	2371	2244	4019	2569	4177	0	0	2956	1723	4609	5382	3204
	0	1531	2761	1140	1819	0	386	10372	4524	0	7309	5746	265	0	0	1785	10139	3715
N1, N3 and Ribosomal spike-ins – 10 copy/reaction	1427	5927	7248	8623	5187	6409	15970	8457	16702	1571	16790	17407	29611	31915	31188	28963	33629	17423
	1961	728	4832	2620	1518	4848	8253	10664	13907	6558	12450	16800	25399	6155	19616	25891	31715	27717
	2981	1224	640	314	278	201	3814	2729	8699	3021	3025	750	897	1211	2111	4601	3563	8436
	1171	1324	0	175	1365	2882	3325	4475	7511	2528	4610	0	1729	3005	3644	3109	9317	5394
	5219	851	1186	5115	2330	2392	8184	4046	5702	6658	5280	7199	9583	7527	7245	3281	6136	5004
	2817	739	3820	1473	3175	3090	9514	4098	7478	6652	7599	7062	3520	9973	10716	9014	7381	8539
	5225	3163	1194	1233	4708	1091	4932	3095	8076	4722	6606	1849	2905	7424	1869	7252	7316	3988
	5408	4084	4353	1018	3049	5050	6408	8881	4638	9093	6337	9860	429	3143	1855	6460	8073	6915

Suppl. Fig. 6. Raw data for limit of detection experiments presented in Fig. 3B. A. Read count table obtained in absence of N1- and N3- control-spike-in. **B.** Read count table obtained in presence of N1- and N3-control-spike-in.

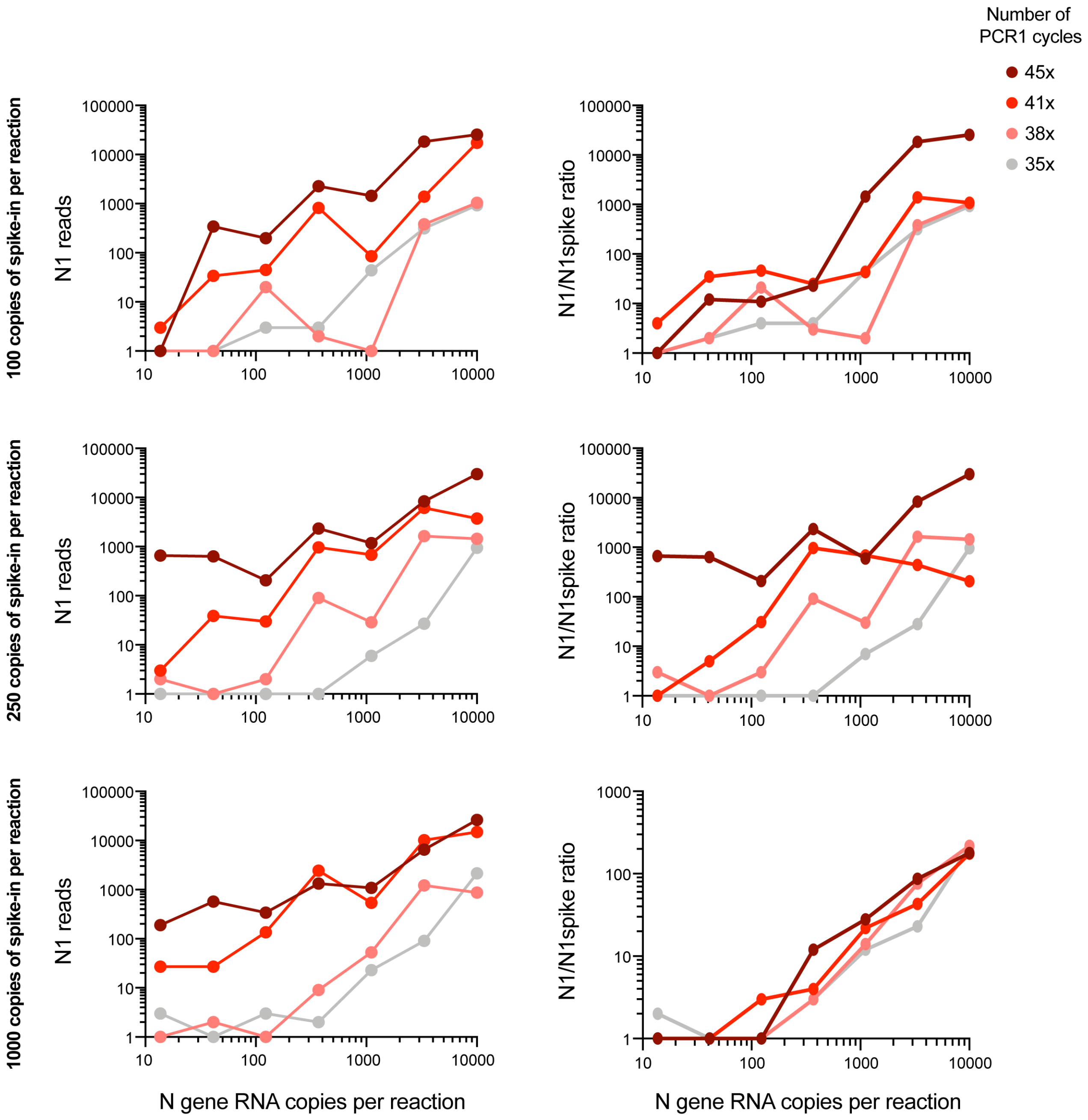

Suppl. Fig. 7. Effects of cycle number and spike-in concentration on sensitivity and quantitativeness. SARSeq was performed in the presence of 100, 250 or 1000 molecules of each N1 and N3 spike-ins (Fig. 3A) as well as 1000 copies of RTC (Suppl. Fig. 3C), on negative gargle samples mixed with QuickExtract and a serial dilution of synthetic SARS-CoV-2 N gene RNA. PCR1 was run for different number of cycles before pooling and running PCR2 for 8 cycles. Both the number of reads for the N1 amplicon, as well as the ratio between N1 and N1 spike-in reads are plotted. The data in the presence of 1000 molecules of spike-ins is also shown in Fig. 3C, D. Spike-in addition did not compromise sensitivity; cycle number on the other hand had the highest effect on sensitivity, as expected. Addition of higher spike-in concentration had a beneficial effect on quantitativeness, most evident when quantifying the ratio of N1/N1 spike, as proposed by the developers of Swab-seq (ref).

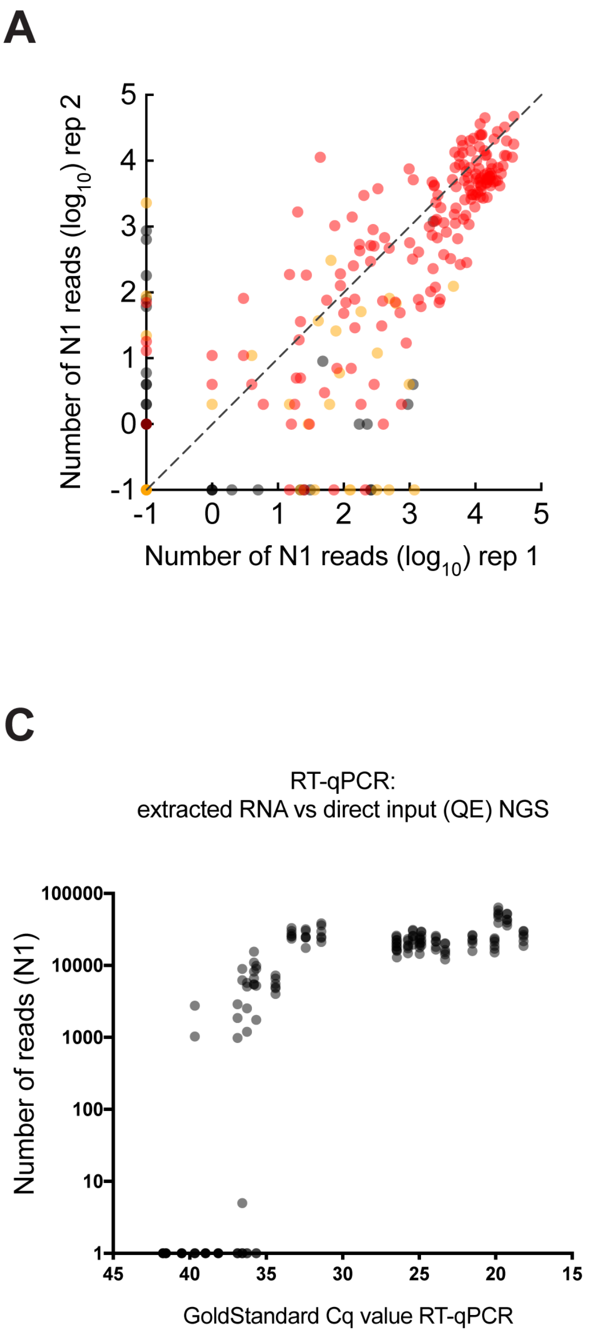

Suppl. Fig. 8. Semiquantitative behaviour of SARSeq on patient samples. A, B. Correlation of read counts of N1 (A) and N3 (B) amplicons across two independent SARSeq runs of the samples shown in Figure 5C, D. In red are individual samples also detected in two qPCR replicates, in orange are samples detected in one out of two qPCR replicates, and in gray those that were not detected by qPCR. **C, D.** Correlation between read counts for N1 (C) and N3 (D) amplicons and Ct values obtained by diagnostic qPCR. The qPCR analysis was performed on purified RNA, in parallel crude samples were measured in seven replicates by SARSeq, all replicates are shown as individual circles. SARSeq is robust until ~Ct 36 and becomes probabilistic in samples with lower viral titers.

A

	gapdh	rRNA	N1	N3	Inf A #1	Inf A #2	Inf B	HRV #1	HRV #2
HEK cells	19,9	8,2						45,5	
HEK + synth. SARS-CoV-2	19,9	8,3	32,0	32,0	46,3		49,4	45,7	
HEK infected Inf A/WSN	20,8	8,2			18,9	23,0	49,3	44,3	
HEK infected Inf A/Wy	21,5	9,3			20,7	25,9	43,0	33,2	40,5
HEK infected Inf B/Lee	20,2	8,9			46,5		28,9	40,8	
HEK infected HRV A1a	20,1	8,0					45,0	16,3	20,9
HEK infected HRV A1b	19,8	8,1						16,7	21,3
HEK infected HRV A2	19,4	7,7			48,8		46,1	11,7	16,3
H_2O		47,4			47,4				

B

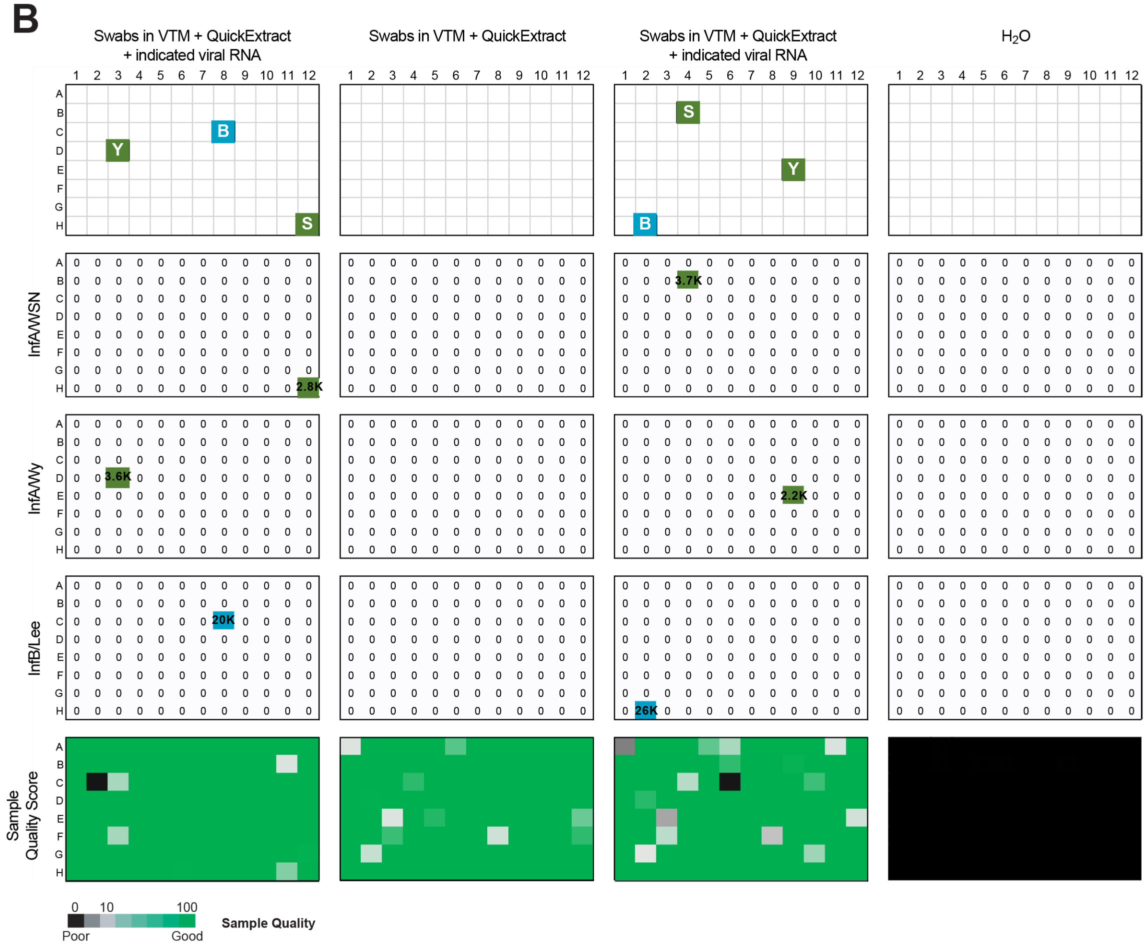

Suppl. Fig. 9. Testing primers for the detection of Influenza and human rhino virus. A. qPCR (using a fluorescent DNA dye Promega) performed on RNA purified from HEK cells infected with Influenza-A/WSN,-A/Wy, Influenza B/Lee, HRV A1a, A1b, or A2. Ct values are shown for respective samples. Primer selection was based on sensitive detection of viral RNA with a low degree of unspecific amplicon generation detected in wells where no specific RNA is expected Primer sequences see Supplemental Table 2. **B.** For Influenza A and Influenza B a full set of 96 primers with well-specific indices was obtained. Clinical samples in VTM, inactivated by heat in QuickExtract were spiked (1:100) with RNA from HEK cells as used above (S: A/WSN; Y: A/Wy; B: B/Lee;). SARSeq was performed with 5 multiplexed primer pairs, N1, N3, rRNA, InfA, and InfB; the two latter and the rRNA/RTC quality score are shown. Analysis of reads allowed for separation of Inf A/WSN and A/Wy based on sequence differences, we also detected Inf B with high specificity. No cross-contamination, index hopping, or assignment ambiguity was observed.

Suppl. Table 1. Guidelines and measures to prevent contamination which could result in false positives.

Possible source of contamination	Measures for minimization
Sample cross contamination	Pipette samples only with filtered tips, exchange gloves after handling sample plates/tubes
	Bleach pipettes, tube racks, plate holders and benches regularly
	Prepare buffers, primer mixes and other reaction master mixes in a clean space in which samples are not handled, if possible, with a separate set of pipettes than the ones used for pipetting samples
Amplicons produced in previous runs, while setting up reactions	Set up physically separated work spaces for pre- and post-amplification steps (not adjacent benches but two different rooms)
	Bleach pipettes, tube racks, plate holders and benches regularly
	Include UTP in all amplification reactions that generate the measured amplicon, being for NGS or other reactions done in the lab. Include the UDG treatment step before each round of amplification to remove possible contaminants from a previous run.
Amplicons sequenced in previous runs	Rotate the different sets of i5/i7 indices between consecutive runs. This way the most likely contaminant (amplicon from the previous run) can be filtered computationally.
	If the same set of i5/i7 indices must be used, record position of highly positive samples and assess whether positive positions from subsequent runs could be due to contamination.
	Wash the sequencer with bleach solution as indicated by the manufacturer. This is particularly important for all flow cells except HiSeq, namely MiSeq, NextSeq500/2000, NovaSeq, iSeq, MiniSeq, where tubing is not exchanged between independent runs.
