# Supplementary material for: Multiplexed detection of SARS-CoV-2 and other respiratory infections in high throughput by SARSeq
Source: Nat Commun. 2021 May 25;12:3132. doi: 10.1038/s41467-021-22664-5 (PMC8149640; doi:10.1038/s41467-021-22664-5)
Supplement: Supplementary file 3 — Description of Additional Supplementary Files [file 41467_2021_22664_MOESM3_ESM.pdf]

Description of Additional Supplementary Files

Title: Supplementary Data 1.
Description: Detailed step-by-step pipetting scheme for every step of the process, from sample preparation to quantifying the library for loading on the sequencer.
Tab 1: Crude RNA preparation by QuickExtract and boiling
Tab 2: Setup of the reverse transcription reaction
Tab 3: Pipetting scheme for the PCR1 Top-up reaction
Tab 4: PCR pooling and Exostar treatment
Tab 5: Pipetting scheme for the setup of PCR2
Tab 6: NGS sample preparation
Tab7: NGS settings for MiSeq system

Title: Supplementary Data 2.
Description: All primer and amplicon sequences used in the study.
Tab 1: A list of all forward primers and barcodes used for SARSeq
Tab 2: A list of all reverse primers and barcodes used for SARSeq
Tab 3: qPCR Primers used to optimize amplicons for Influenza and HRV
Tab 4: Sequences of all virus-specific amplicons detected in this manuscript
Tab 5: Oligonucleotides used to clone the T7 transcribed internal control without generating templates that interfere with primer synthesis regulations.
