# Supplementary material for: Multiplexed detection of SARS-CoV-2 and other respiratory infections in high throughput by SARSeq
Source: Nat Commun. 2021 May 25;12:3132. doi: 10.1038/s41467-021-22664-5 (PMC8149640; doi:10.1038/s41467-021-22664-5)
Supplement: Supplementary file 6 — Reporting summary [file 41467_2021_22664_MOESM6_ESM.pdf]

nature research

Corresponding author(s): Luisa Cochella and Ulrich Elling

Last updated by author(s): Mar 16, 2021

Reporting Summary

Nature Research wishes to improve the reproducibility of the work that we publish. This form provides structure for consistency and transparency in reporting. For further information on Nature Research policies, see our Editorial Policies and the Editorial Policy Checklist.

Statistics

For all statistical analyses, confirm that the following items are present in the figure legend, table legend, main text, or Methods section.

n/a	Confirmed	
☐	☒	The exact sample size (*n*) for each experimental group/condition, given as a discrete number and unit of measurement
☐	☒	A statement on whether measurements were taken from distinct samples or whether the same sample was measured repeatedly
☒	☐	The statistical test(s) used AND whether they are one- or two-sided<br>*Only common tests should be described solely by name; describe more complex techniques in the Methods section.*
☒	☐	A description of all covariates tested
☒	☐	A description of any assumptions or corrections, such as tests of normality and adjustment for multiple comparisons
☐	☒	A full description of the statistical parameters including central tendency (e.g. means) or other basic estimates (e.g. regression coefficient) AND variation (e.g. standard deviation) or associated estimates of uncertainty (e.g. confidence intervals)
☒	☐	For null hypothesis testing, the test statistic (e.g. *F*, *t*, *r*) with confidence intervals, effect sizes, degrees of freedom and *P* value noted<br>*Give P values as exact values whenever suitable.*
☒	☐	For Bayesian analysis, information on the choice of priors and Markov chain Monte Carlo settings
☒	☐	For hierarchical and complex designs, identification of the appropriate level for tests and full reporting of outcomes
☒	☐	Estimates of effect sizes (e.g. Cohen's *d*, Pearson's *r*), indicating how they were calculated

Our web collection on statistics for biologists contains articles on many of the points above.

Software and code

Policy information about availability of computer code

Data collection	Illumina MiSeq V2, NextSeq Mid Output, NextSeq High Output, BioRad qPCR CFX Connect and software CFX Maestro;
Data analysis	Custom code was used to analyze all NGS data. The script is available on GitHub at https://github.com/alex-stark-imp/SARSeq and at https://starklab.org. Plots were generated using Graphpad Prism 8.4.3.

For manuscripts utilizing custom algorithms or software that are central to the research but not yet described in published literature, software must be made available to editors and reviewers. We strongly encourage code deposition in a community repository (e.g. GitHub). See the Nature Research guidelines for submitting code & software for further information.

Data

Policy information about availability of data

All manuscripts must include a data availability statement. This statement should provide the following information, where applicable:

- Accession codes, unique identifiers, or web links for publicly available datasets
- A list of figures that have associated raw data
- A description of any restrictions on data availability

Sequencing data for all experiments are available at GEO, under accession number GSE163688. Figures with associated raw data are: 1D,E, 2, 3B-E, 4A-H, 5, S1C, S1D, S2B, S3, S4, S5, S6, S7, S8. All sequencing data is made available, all primer sequences are submitted as Supplemental Table 2.

Field-specific reporting

Please select the one below that is the best fit for your research. If you are not sure, read the appropriate sections before making your selection.

☒ Life sciences ☐ Behavioural & social sciences ☐ Ecological, evolutionary & environmental sciences

For a reference copy of the document with all sections, see nature.com/documents/nr-reporting-summary-flat.pdf

Life sciences study design

All studies must disclose on these points even when the disclosure is negative.

Sample size	This manuscript does not generate conclusions based on statistical analysis, that is why sample size calculations were not necessary. We analyzed many samples, each individually, for the Sars-CoV2 status. Every set of experiment was performed on at least 192 individual samples (typically more), which is in excess of what is necessary to conclude specificity and sensitivity parameters on positive and negative samples.
Data exclusions	Of experiments presented all replicates and data points are shown, no data were excluded. For clarity of the manuscript, preliminary experiments used to set up the method are not all shown.
Replication	Results of replicate analysis are presented in Fig. 4A-F and G-H. No replicates excluded. In cases where multiple samples were measured in one experiment, to show reproducibility of successful detection, all samples are shown. All attempts at replication were successful
Randomization	No experimental groups. Where feasible, positive samples were spiked in random locations in input, see experimental layout.
Blinding	The experimenters preparing sample plates were not blinded. However, the analysis of every sample is purely objective (sequencer and computational analysis, or qPCR), therefore no blinding was necessary at that step. The bioinformatic analysis was blind to the status of sample input.

Reporting for specific materials, systems and methods

We require information from authors about some types of materials, experimental systems and methods used in many studies. Here, indicate whether each material, system or method listed is relevant to your study. If you are not sure if a list item applies to your research, read the appropriate section before selecting a response.

Materials & experimental systems

n/a	Involved in the study
☒	☐ Antibodies
☐	☒ Eukaryotic cell lines
☒	☐ Palaeontology and archaeology
☒	☐ Animals and other organisms
☐	☒ Human research participants
☒	☐ Clinical data
☒	☐ Dual use research of concern

Methods

n/a	Involved in the study
☒	☐ ChIP-seq
☒	☐ Flow cytometry
☒	☐ MRI-based neuroimaging

Eukaryotic cell lines

Policy information about cell lines

Cell line source(s)	HEK293T Lenti-X cells (TaKaRa) were used only as a source of human ribosomal RNA and infection with influenza to obtain RNA.
Authentication	Cell line was purchased directly from provider, expanded and frozen at passage 3. Early passages of this stock collection were used for all experiments. Cell line was not further authenticated.
Mycoplasma contamination	Mycoplasma status is monitored routinely (weekly). No mycoplasma was detected.
Commonly misidentified lines (See ICLAC register)	No commonly misidentified cell lines were used in the study.

Human research participants

Policy information about studies involving human research participants

Population characteristics	No covariant data were collected, all samples were processed blinded to personal data, no statistical analysis was performed on human populations.
Recruitment	Self-selection bias is not relevant for this study. Samples were anonymously collected in an in-house monitoring pipeline as well as in clinical settings. For in house testing, all employees are routinely tested. For samples collected in clinical setting these were primarily of COVID-19 symptomatic individuals, or individual subject to contact tracing, or COVID-19 negative individuals that required testing for other reasons. All samples were annonymized to the experimenters.
Ethics oversight	The present study includes preliminary investigations and results of a clinical performance study approved by the local Ethic Committee of Vienna (#EK 20-208-0920). For that, left-over samples from healthy participants were obtained from an anonymous routine SARS-CoV-2 screening pipeline, and left-over patient samples in Fig. 4G, H were obtained by the Austrian Agency for Health and Food Safety (AGES) in a diagnostic pipeline and provided to us fully anonymized. For VTM samples used for Figure 4A-F, an additional approval (#06-04-9-33163 from 21/07/2020) was obtained from the Ethics Committee of the Clinical Center of the University of Sarajevo. The study was conducted in accordance with the Declaration of Helsinki.

Note that full information on the approval of the study protocol must also be provided in the manuscript.
